# Kindlin-3 interacts with the ribosome and regulates c-Myc expression required for proliferation of chronic myeloid leukemia cells

**DOI:** 10.1038/srep18491

**Published:** 2015-12-18

**Authors:** Jing Qu, Rya Ero, Chen Feng, Li-Teng Ong, Hui-Foon Tan, Hui-Shan Lee, Muhammad HB Ismail, Wen-Ting Bu, Srikanth Nama, Prabha Sampath, Yong-Gui Gao, Suet-Mien Tan

**Affiliations:** 1 School of Biological Sciences, Nanyang Technological University, 60 Nanyang Drive, Singapore 637551, Singapore; 2 Institute of Medical Biology, 8A Biomedical Grove, Singapore 138648, Singapore; 3 Department of Biochemistry, Yong Loo Lin School of Medicine, National University of Singapore 117597,Singapore; 4 Program in Cancer and Stem Cell Biology, Duke-NUS Graduate Medical School, Singapore 169857, Singapore; 5 Institute of Molecular and Cell Biology, 61 Biopolis Drive, Proteos, Singapore 138673, Singapore

## Abstract

Kindlins are FERM-containing cytoplasmic proteins that regulate integrin-mediated cell-cell and cell-extracellular matrix (ECM) attachments. Kindlin-3 is expressed in hematopoietic cells, platelets, and endothelial cells. Studies have shown that kindlin-3 stabilizes cell adhesion mediated by ß1, ß2, and ß3 integrins. Apart from integrin cytoplasmic tails, kindlins are known to interact with other cytoplasmic proteins. Here we demonstrate that kindlin-3 can associate with ribosome via the receptor for activated-C kinase 1 (RACK1) scaffold protein based on immunoprecipitation, ribosome binding, and proximity ligation assays. We show that kindlin-3 regulates c-Myc protein expression in the human chronic myeloid leukemia cell line K562. Cell proliferation was reduced following siRNA reduction of kindlin-3 expression and a significant reduction in tumor mass was observed in xenograft experiments. Mechanistically, kindlin-3 is involved in integrin α5ß1-Akt-mTOR-p70S6K signaling; however, its regulation of c-Myc protein expression could be independent of this signaling axis.

Kindlins are a small family of 4.1-ezrin-radixin-moesin (FERM)-containing cytoplasmic proteins that regulate integrin activation and outside-in signaling^1^,^2^,^3^,^4^. Kindlin-1, -2, and -3 have distinct but overlapping expression patterns^5^,^6^. They have non-redundant functions as exemplified by specific diseases associated with each paralog. The skin fragility disorder Kindler’s syndrome is ascribed to mutations in kindlin-1^7^. Kindlin-2 is involved in cancer progression and its deficiency is embryonic lethal^8^,^9^. Deficiency in kindlin-3 is the cause of Leukocyte Adhesion Deficiency III characterized by defective platelet coagulation and leukocyte migration^10^. All kindlins contain an N-terminal F0 domain and C-terminal FERM domain linearly organized into regions: F1, F2 bisected by a pleckstrin homology (PH) domain, and F3^11^. Kindlins bind to the membrane distal NxxY/F motif of the ß integrin cytoplasmic tails^10^,^12^. Together with talin, they positively regulate integrin ligand-binding avidity^13^,^14^. Kindlins are also involved in mitotic spindle assembly, clathrin-mediated endocytosis, Wnt-signaling, and assembly of the erythrocyte membrane-cytoskeleton^8^,^15^,^16^,^17^,^18^.

Kindlin-3 is expressed in osteoclasts, platelets, hematopoietic and endothelial cells^5^,^6^,^19^,^20^. In addition to leukocyte migration and platelet aggregation, kindlin-3 is involved in endothelial tube formation and osteoclast-mediated bone resorption^6^,^20^. Recently, kindlin-3 was found to be important in cancer progression although its role as a promoter or suppresser of cancer metastasis remains controversial^21^,^22^. Non-integrin binding partners of kindlin-3 have been identified. In platelets, kindlin-3 associates with the adhesion and degranulation promoting adaptor protein (ADAP) and, together with talin, promotes the activation of integrin αIIbß3^23^. We reported previously the association of kindlin-3 with the receptor for activated-C kinase 1 (RACK1)^24^.

RACK1 is ubiquitously expressed in all tissues and it is a Trp-Asp (WD) 40 ß-propeller cytoplasmic protein^25^,^26^. It has many binding partners, including activated protein kinase C (PKC), c-Src, G protein ßγsubunits, as well as ß1, ß2, and ß5 integrin cytoplasmic tails^27^,^28^,^29^,^30^. RACK1 localizes to nascent focal complexes but not to mature focal adhesions^31^,^32^. RACK1 forms a complex with focal adhesion kinase (FAK) and phosphodiesterase 4D5 (PDE4D5) that mediates direction sensing in migrating cells^33^. RACK1 is also a core component of the eukaryotic 40S ribosome subunit and it regulates protein translation under physiological and pathological conditions^25^,^34^,^35^,^36^. Recently, it has been shown to promote internal ribosome entry site (IRES)-mediated translation of hepatitis C viral proteins^37^.

In this study, we report the novel observation of kindlin-3 associating with ribosome through RACK1. This association was detected in hematopoietic cell lines and human umbilical vein endothelial cells (HUVECs). Further, we show that silencing kindlin-3 expression in the chronic myeloid leukemic cell line K562 reduced c-Myc protein expression, suggesting a role for kindlin-3 in regulating c-Myc protein synthesis. Consistent with these findings, silencing of kindlin-3 expression significantly reduced K562 tumor growth in mouse xenograft model. Although kindlin-3 is involved in fibronectin-engaged integrin α5ß1-Akt-mTOR-p70S6K signaling in K562 cells, our data suggest that kindlin-3 regulates c-Myc protein expression by a pathway that could be independent of this signaling axis.

## Results

### Kindlin-3 associates with ribosomes

**Figure 1:**
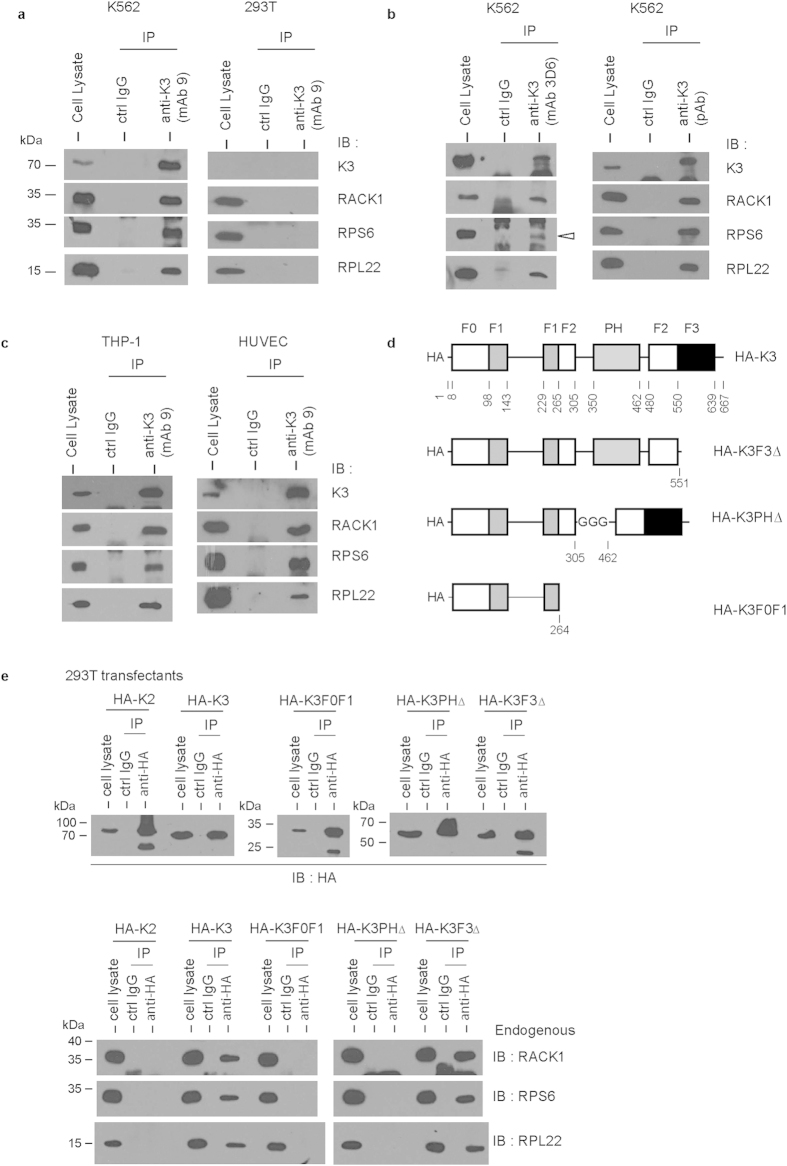
Co-immunoprecipitation assays of kindlin-3. (**a**) K562 and 293T cells were lysed and immunoprecipitation was performed using anti-kindlin-3 mAb clone 9^24^. Rat IgG was used as the control antibody (ctrl IgG). Co-precipitated RACK1, RPS6, and RPL22 were detected by Western blotting. (**b**) Immunoprecipitation of kindlin-3 using alternative antibodies, namely the previously reported^38^ mAb 3D6 and pAb from commercial source (Sigma). Mouse IgG and rabbit IgG were used as ctrl IgGs, respectively. (**c**) Immunoprecipitation of kindlin-3 from cell lysates of THP-1 and HUVEC. (**d**) HA-tagged kindlin-3 expression constructs used in this study. HA-K3 (full-length kindlin-3), HA-K3F3Δ (kindlin-3 with F3 subdomain deletion), HA-K3PHΔ (kindlin-3 with PH domain deletion and a triple-Gly linker insertion between the two F2-subdomains), HA-K3F0F1 (kindlin-3 truncation containing only the F0-F1 regions). (**e**) Co-immunoprecipitation assays using 293T cells transfected with the indicated HA-tagged kindlin expression constructs.

Kindlin-3 was immunoprecipitated from K562 cell lysate using anti-kindlin-3 mAb (clone 9)^24^. RACK1, 40S ribosomal protein RPS6, and 60S ribosomal protein RPL22, were detected in the co-precipitate by immunoblotting (Fig. 1a). We ruled out the possibility of non-specific interactions as these ribosomal proteins were not detected in immunoprecipitation samples using the same mAb but with cell lysate of human kidney fibroblast 293T that does not express kindlin-3. These data suggest that kindlin-3 specifically associates with ribosomes.

To further verify these observations, kindlin-3 was immunoprecipitated from K562 cell lysate using two additional anti-kindlin-3 antibodies, the mAb 3D6 reported previously^38^ and a pAb from commercial source (Fig. 1b). RACK1, RPS6, and RPL22 were detected in the co-precipitates of these antibodies. The association of kindlin-3 with ribosome is not cell line-specific since similar results were obtained when kindlin-3 immunoprecipitation was performed on cell lysates of kindlin-3-expressing monocytic THP-1 cell line and HUVECs (Fig. 1c).

Kindlin-3 PH domain has been shown to be required for binding to RACK1^24^. 293T cells were transfected with HA-tagged full-length kindlin-3, its truncated mutants or HA-tagged full-length kindlin-2 (Fig. 1d). The truncated kindlin-3 mutants were K3F3Δ (F3 sub-domain deletion), K3PHΔ (PH domain deletion with a triple-Gly insertion) and K3F0F1 (deletion after the F0-F1 sub-domains). Ectopically expressed kindlins were immunoprecipitated using anti-HA antibody followed by immunoblotting (Fig. 1e). RACK1, RPS6, and RPL22 were detected in the co-precipitate of full-length kindlin-3 but not kindlin-2. F3 subdomain deletion did not impair the association of kindlin-3 with the RACK1-ribosome complex. However, the association was abrogated as a result of PH domain deletion. Conceivably, K3F0F1 failed to associate with RACK1-ribosome complex.

### Verification of the association of kindlin-3 with RACK1-ribosome complex by ribosome fractionation and *in vitro* ribosome-binding experiments

**Figure 2:**
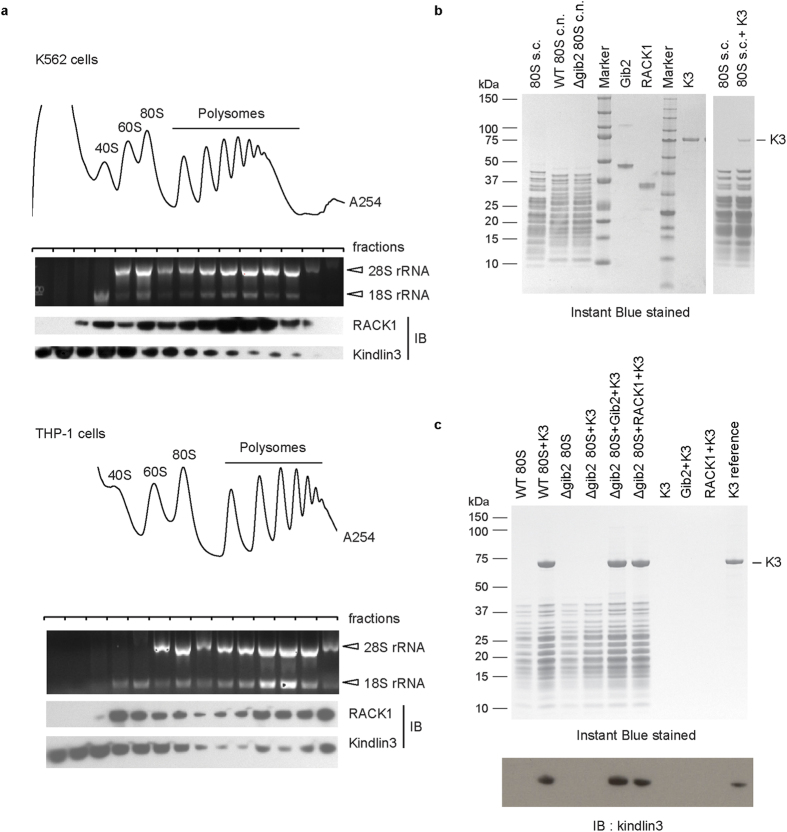
Kindlin-3 associates with ribosomes as determined by sucrose density gradient fractionation and ribosome binding assays. (**a**) Ribosome fractionation using K562 and THP-1 cell lysates subjected to sucrose density gradient centrifugation and fractionation. Ribosome profile at A_254_ and the corresponding fractions are shown. 28S and 18S rRNAs were resolved by agarose gel electrophoresis. RACK1 and kindlin-3 in each fraction were detected by Western blotting. Representative data are shown. (**b**,**c**) *In vitro* binding assay using recombinant kindlin-3 and 80S ribosomes isolated from *S. cerevisiae* (s.c.) and *C. neoformans* (c.n.) wild-type as well as *C. neoformans* Gib2-deficient (c.n. Δgib2) strains. (**b**) *In vitro* binding experiment using s.c. 80S ribosome and purified kindlin-3 (right panel). Kindlin-3 was incubated with s.c. 80S ribosome and sedimented through sucrose cushion by high speed centrifugation Proteins of sedimented pellet were resolved by SDS-PAGE. SDS-PAGE of recombinant Gib2, RACK1, and kindlin-3 proteins is shown as control (left panel). (**c**) *In vitro* binding assay using c.n. wild-type and Δgib2 80S ribosomes and purified recombinant kindlin-3 with or without RACK1 or Gib2. For control, proteins were incubated without ribosomes. Binding assay was carried out as in (**b**). Instant Blue stained gel (top panel) and kindlin-3 immunoblot (IB) (bottom panel) are shown.

Whole cell lysates of K562 and THP-1 were subjected to sucrose density gradient fractionation. Ribosome profile was determined by A_254_ measurement and 28S and 18S rRNA (major components of ribosome 60S and 40S subunits, respectively) distribution in the fractions was analyzed by agarose gel electrophoresis (Fig. 2a). The presence of RACK1 and kindlin-3 in the fractions was determined by immunoblotting. In accordance with being a core component of the 40S subunit, a high level of RACK1 was detected in fractions corresponding to 40S subunits, 80S ribosomes (monosomes), and polysomes (two or more ribosomes bound to same mRNA). Kindlin-3 was detected in these fractions but at a higher level in fractions containing the monosomes than the polysomes.

*In vitro* ribosome binding assays were performed to determine if kindlin-3 binding to ribosome is RACK1-dependent. The RACK1 orthologue Asc1 (53% sequence identity) in *Saccharomyces cerevisiae* (budding yeast) adopts a seven-bladed ß propeller structure^25^ and it is a core component of *S. cerevisiae* 40S subunit^39^. 80S ribosomes were purified from *S. cerevisiae* as described previously^40^ and the full-length N-terminal His_6_-tagged kindlin-3 was purified using an insect-cell expression system. Both components were resolved by SDS-PAGE and visualized by Instant Blue staining (Fig. 2b). 80 S ribosomes were incubated with kindlin-3 and pelleted through sucrose cushion by centrifugation. Kindlin-3 was detected in the ribosomal pellet (Fig. 2b, right panel). The association is most likely mediated by Asc1co-purifying with ribosomes serving as the bridging molecule.

The RACK1 orthologue expressed in the fungus *Cryptococcus neoformans* is Gib2 (70% sequence identity)^41^, and the two proteins share similar overall structures^42^. Unlike mammalian RACK1 and yeast Asc1, Gib2 is not an essential protein because the *gib2* gene deletion strain of *C. neoformans* has been reported^43^. To demonstrate that RACK1 serves as the bridging molecule in complex formation between kindlin-3 and ribosome, 80S ribosomes were purified from wild-type *C. neoformans* and *gib2* deletion (Δ*gib2*) mutant strain. Recombinant Gib2 and RACK1 (both with an N-terminal His_6_-tag) were expressed in *E. coli* and purified. The ribosome preparations, as well as the recombinant Gib2 and RACK1 were verified by SDS-PAGE (Fig. 2b). These preparations together with the recombinant kindlin-3 were used in ribosomal binding assays as described above for *S. cerevisiae* studies. Kindlin-3 was detected in the wild-type but not the Δ*gib2* 80S ribosome pellet (Fig. 2c), suggesting that the endogenous Gib2 co-purifying with wild-type ribosomes acts as the link between kindlin-3 and the ribosome. In accordance, kindlin-3 was detected in the Δ*gib2* 80S ribosome pellet in the presence of recombinant Gib2 as well as RACK1. Collectively, these data suggest that RACK1 serves as the bridging molecule for the association between kindlin-3 and the ribosome.

### Detecting kindlin-3-RACK1-ribosome complex in HUVECs

**Figure 3:**
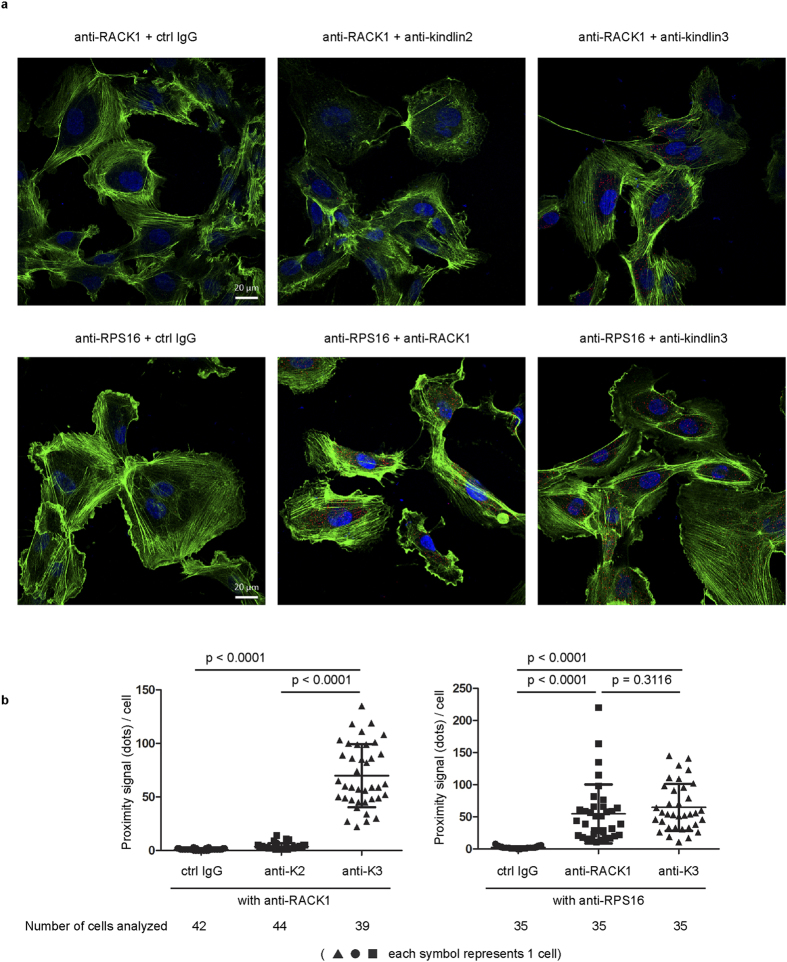
Proximity ligation assay (PLA) to detect kindlin-3 and RACK1-ribosome in HUVECs. (**a**) PLA was performed on HUVECs growing on uncoated coverslip glass-bottom culture dish using relevant antibodies as indicated. Proximity ligation signal (red dots in cells) and actin filaments (stained with Alexa-Fluor®488-conjugated phallodin) were examined under confocal laser scanning microscope. Scale bar, 20 μm. (**b**) Confocal microscope images were analyzed using the Blobfinder software to determine the number of red dots per cell and results were plotted. Two-tailed unpaired *t* test was performed. Data shown are from one experiment. The second independent experiment is shown in Supplementary Fig. S1.

To confirm the results of our co-immunoprecipitation and *in vitro* ribosome-binding assays, we examined the association of kindlin-3-RACK1-ribosome in cells using the proximity ligation assay (PLA). Instead of K562 cells, we chose to analyze HUVECs because they are adherent with a large cell-spread area. Further, HUVECs express both kindlin-3 and kindlin-2 which is useful for the determination of specificity. PLAs were performed on cells that were cultured on uncoated coverslip-bottom culture dishes. Significant number of proximity signals (dots) per cell was detected in sample incubated with anti-RACK1 and anti-kindlin-3 antibodies but not in samples treated with anti-RACK1 and anti-kindlin-2 or control IgG antibodies (Fig. 3a,b; supplementary Fig. S1), suggesting that kindlin-3, but not kindlin-2, is in close proximity with RACK1. In order to verify that kindlin-3 is proximal to the ribosome, PLA was also performed to detect RPS16 (ribosomal protein in the small subunit) with kindlin-3 and RACK1. Based on the crystal structure of the 80S ribosome^40^, the distance between RPS16 and RACK1 is <40 nm, which is within the theoretical maximum distance between two target proteins required for PLA detection. Hence, the association of kindlin-3 with RACK1 should position kindlin-3 in the vicinity of RPS16 within the working distance of PLA. Indeed, we detected significant proximity signal in samples treated with anti-RPS16 and anti-kindlin-3 or anti-RACK1 compared with control IgG (Fig. 3a,b; supplemental Fig. S1).

### c-Myc protein expression is down-regulated in K562 cells expressing kindlin-3-targeting siRNA

**Figure 4:**
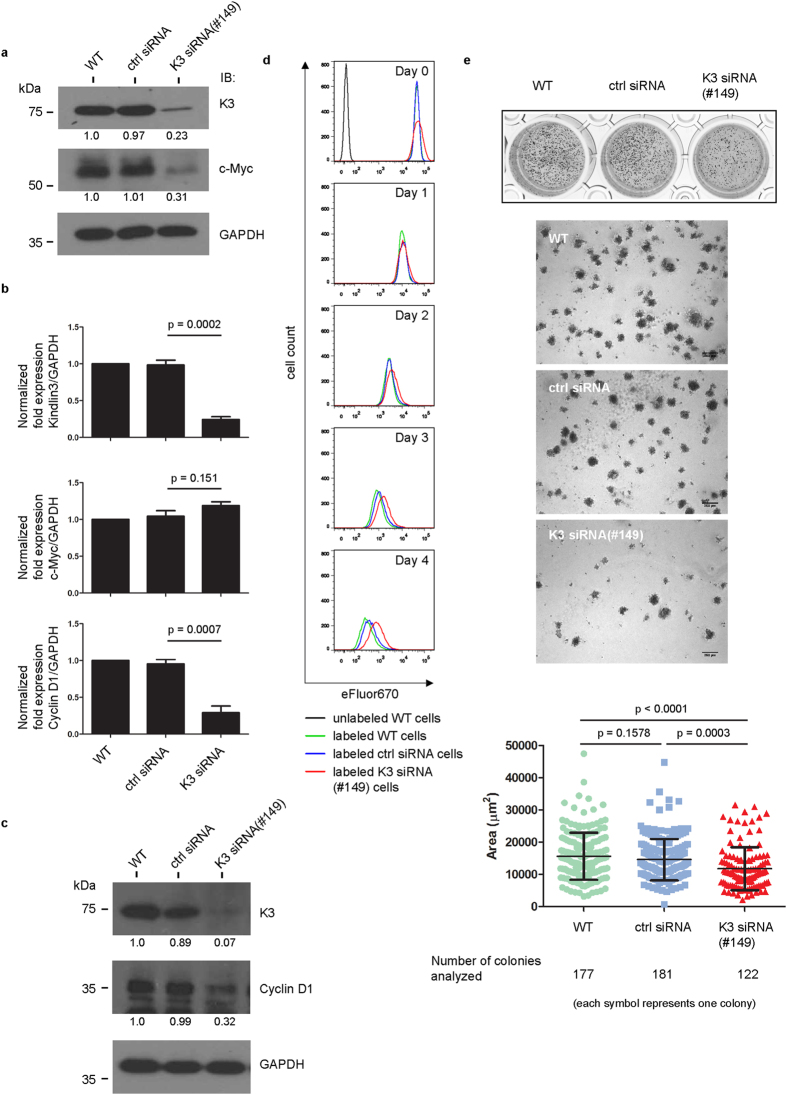
Kindlin-3 regulates c-Myc protein expression in K562 cells. (**a**) Western blot analyses of c-Myc expression in wild-type, control siRNA, and kindlin-3 siRNA(#149) cells. GAPDH serves as loading control. Values below protein bands represent the mean fold differences in protein expression levels relative to WT samples (normalized to 1.0) from three independent experiments. (**b**) RT-qPCR analyses of mRNA expression of kindlin-3, c-Myc and cyclin D1. Values represent mean ± S.E.M. of three independent experiments. Wild-type mean value was normalized to 1.0. Two-tailed unpaired *t* test was performed to compare control siRNA cells with kindlin-3 siRNA(#149) cells. p < 0.05 is considered significant. (**c**) Western blot of cyclin D1 in these cells. Mean fold differences from three independent experiments were determined as in (**a**). (**d**) Cell proliferation rate determination using cell proliferation dye eFluor® 670 and flow cytometry analyses. A representative experiment of three independent experiments is shown. (**e**) Soft agar colony formation assay. Cells were stained with NBT for clearer visualization. Representative microscope images of wells are shown. Scale bar, 250 μm. The area of each tumor colony was determined and plotted. A combined plot of tumor colonies from two independent experiments is shown. Two-tailed unpaired *t* test was performed. p < 0.05 is considered significant.

The association of kindlin-3 with ribosomes prompted us to ask whether kindlin-3 has a role in modulating protein translation. The transcription factor c-Myc, which regulates cell cycle progression and apoptosis, is deregulated in many forms of cancer^44^. Interestingly, it has been reported that RACK1 positively regulates c-Myc protein translation in hepatocellular carcinoma^34^. Our data suggest that kindlin-3 interacts with the RACK1-ribosome complex, hence we extended our study to examine whether kindlin-3 plays a role in regulating c-Myc protein translation. c-Myc protein expression was markedly reduced in cells expressing kindlin-3-targeting siRNA(#149) but not control siRNA or in wild-type cells (Fig. 4a). However, c-Myc mRNA expression levels were comparable amongst all three groups of cells (Fig. 4b). These data suggest that silencing of kindlin-3 expression in K562 cells down-regulates c-Myc protein expression, but not c-Myc mRNA expression. c-Myc regulates cyclin D1 expression^44^. Hence, we examined the expression level of cyclin D1 in these cells (Fig. 4b,c). Both protein and mRNA expression levels of cyclin D1 were reduced in K562 cells expressing kindlin-3 siRNA(#149). We then examined the proliferation rate of these cells in suspension culture. Cells were labeled with proliferation dye eFluor670 and analyzed by flow cytometry. Fluorescence intensity of cells decreases with time because incorporated dye is apportioned into daughter cells upon cell division. The fluorescence intensity of K562 cells expressing kindlin-3 siRNA(#149) decreased at a slower rate compared with other cells (Fig. 4d), suggesting that silencing kindlin-3 expression reduces proliferation rate. We repeated the above experiments using another kindlin-3 siRNA (#758) and data obtained were consistent (Supplementary Fig. S2). This excludes the possibility of off-target effects. We also performed rescue experiments by overexpressing siRNA-resistant HA-kindlin-3 or HA-kindlin-3 PHΔ in kindlin-3 siRNA(#149) K562 cells (Supplementary Fig. S3). The expression levels of HA-kindlin-3 and HA-kindlin-3 PHΔ were verified by RT-qPCR and immunoblotting. c-Myc expression was rescued in cells expressing HA-kindlin-3 but to a lesser extent in cells expressing HA-kindlin-3 PHΔ. These data verify a role of kindlin-3 in regulating c-Myc expression. This could be dependent on kindlin-3/RACK1 interaction because we have shown in previous section that PH domain deletion disrupts kindlin-3 and RACK1 binding. We also performed soft agar colony formation assay. Cells were embedded in soft agar and allowed to grow and form colonies. Consistent with the above data, we observed smaller colonies of cells expressing kindlin-3 siRNA(#149) compared with control siRNA or wild-type cells (Fig. 4e).

### Kindlin-3 silencing reduced K562 tumor size in xenograft models

**Figure 5:**
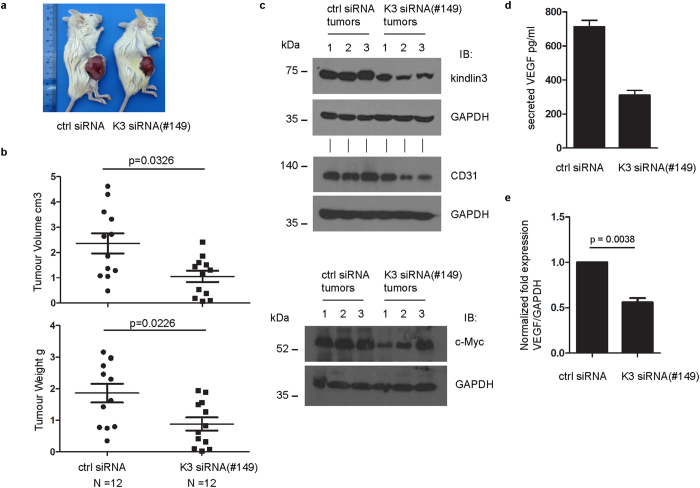
Xenotransplant tumor formation study. (**a**) Representative image of tumor formation in Balb/c-Rag−/−IL2Rγ−/− mice transplanted with either control siRNA or kindlin-3 siRNA(#149) K562 cells. (**b**) Plots of tumor volume (cm^3^) and weight (g). N = 12. Each closed circle or square represents one mouse. Note: there are two overlapping dots for the control siRNA group in the tumor-weight plot. Mann-Whitney test (two-tailed) was performed. p < 0.05 is considered significant. (**c**) Western blot analyses of CD31 and c-Myc expression in tissue samples excised from tumors. Three tumors were examined from each group. GAPDH serves as loading control. Kindlin-3 expression in these tumors was also examined. (**d**) Secreted VEGF-A level in culture supernatant of cells as determined by ELISA. A representative experiment of two independent experiments is shown. Data are means +/− S.D. of technical triplicates. (**e**) RT-qPCR determination of VEGF-A mRNA levels in the two groups of cells. Values represent mean ± S.E.M. of three independent experiments. Wild-type mean value was normalized to 1.0. Two-tailed paired *t* test was performed to compare control siRNA cells with kindlin-3 siRNA(#149) cells. p < 0.05 is considered significant.

We extended the study to investigate the effect of kindlin-3 silencing on tumor formation *in vivo*. K562 cells expressing either control siRNA or kindlin-3-siRNA(#149) were transplanted into the right flank of Balb/c-Rag−/−IL2Rγ−/− mice. Kindlin-3-siRNA(#149) cells formed significantly smaller (volume and weight) tumors compared to control siRNA cells (Fig. 5a,b). Tumor formation is highly dependent on angiogenesis and vascularization. Reduced expression of CD31 (PECAM-1), a surface antigen found on endothelial cells, was detected in kindlin-3 siRNA tumors compared with control siRNA tumors (Fig. 5c), suggesting poor vascularization in the former. Further, consistent with our *in vitro* data, kindlin-3-siRNA(#149) tumors expressed lower levels of c-Myc compared with control siRNA tumors (Fig. 5c). Vascular endothelial growth factor (VEGF) promotes angiogenesis and vascularization. We therefore examined the expression level of VEGF-A in kindlin-3-siRNA(#149) cells and control siRNA cells. Compared with control siRNA cells, the levels of secreted VEGF-A and its mRNA were markedly reduced in kindlin-3-siRNA(#149) cells (Fig. 5d,e).

### Kindlin-3 regulates integrin α5ß1-derived Akt-mTOR-p70S6K signaling but this pathway is not critical for its role in regulating c-Myc protein expression

**Figure 6:**
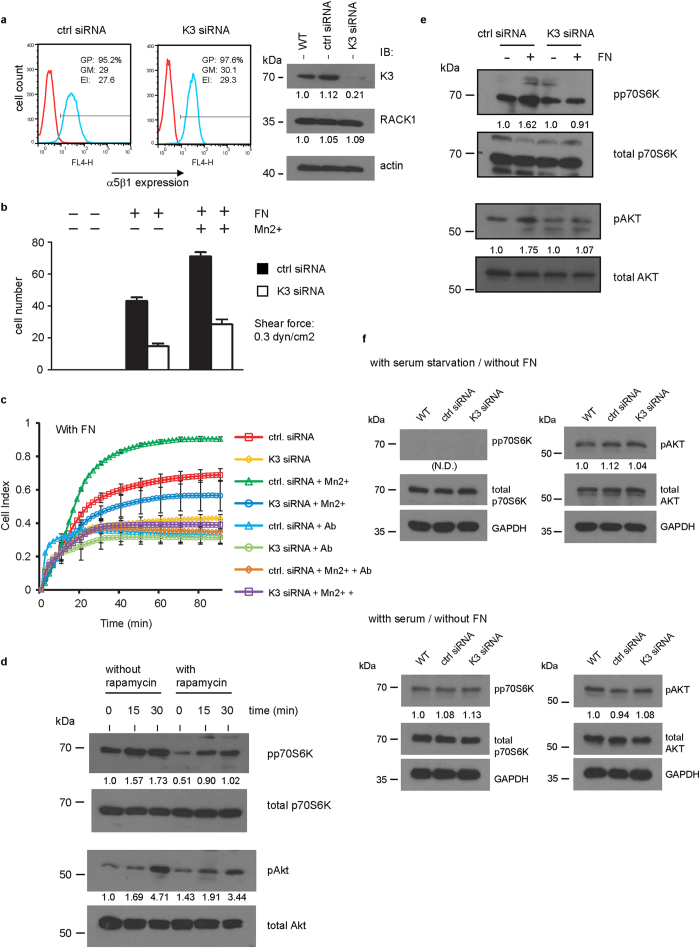
Analyses of the role of kindlin-3 in integrin α5ß1 mediated cell adhesion and activation of Akt-mTOR-p70S6K signaling pathway. (**a**) (left panel) Flow cytometry analyses of integrin α5ß1 expression in K562 cells expressing either control siRNA or kindlin-3-siRNA(#149). GP: % gated positive; GM: geo-mean fluorescence; EI: expression index = % GP x GM. (right panel) Western blot analysis of kindlin-3 and RACK1 in these cells. Actin serves as loading control. Values below protein bands represent the mean fold differences in protein expression levels relative to WT samples (normalized to 1.0) from three independent experiments. (**b**) Flow assay of cells on immobilized fibronectin (FN) at a shear stress of 0.3 dyn/cm^2^. Data point represents mean of cell number of four different fields analyzed. Representative plots of two independent experiments are shown. (**c**) ECIS measurements of cell adhesion and spreading on immobilized fibronectin. Representative plots of two independent experiments of cell spreading (represented by cell index – impedance readout) against time are shown. Data point represents the mean ± S.D. of technical triplicates. MnCl_2_ and function-blocking anti-integrin α5 antibody (Ab) were included. (**d–f**) Western blot analyses of p70S6K and pAkt and their phosphorylated forms under different conditions indicated. The end concentrations of reagents used are: rapamycin (1 μM), soluble fibronectin (20 μg/ml), and MnCl_2_ (1 mM). Values below protein bands represent the mean fold differences of phosphor-protein/total protein relative to either WT sample or untreated sample (normalized to 1.0) from three independent experiments. N.D.: Not determined because phosphor-protein band was not detected.

Kindlins are important regulators of integrin functions. K562 cell line expresses high level of fibronectin receptor integrin α5ß1 but not integrin αVß3^45^. It is therefore possible that kindlin-3 is involved in integrin α5ß1 signaling that regulates the mTOR signal pathway. First, we determined whether or not kindlin-3 is required for integrin α5ß1 ligand-binding. Shear flow assays showed that kindlin-3-siRNA(#149) K562 cells adhered poorly to fibronectin in the presence of exogenous activating agent Mn^2+^ compared with cells expressing control siRNA, although the two groups of cells expressed comparable levels of integrin α5ß1 and RACK1 (Fig. 6a,b). These results were verified by real-time measurements of cell adhesion and spreading on fibronectin using the electrical cell-substrate impedance sensing (ECIS) system (Fig. 6c). We then determined the activities of Akt and p70S6K in these cells because these kinases are involved in the mTOR signaling pathway. An increase in the activation of both kinases, which could be blunted by mTORc inhibitor rapamycin, was detected in wild-type K562 cells treated with fibronectin (Fig. 6d). The level of activation of these kinases in kindlin-3-siRNA(#149) cells in the presence of fibronectin was reduced compared with control siRNA cells (Fig. 6e). These data suggest that kindlin-3 is involved in integrin α5ß1 activation of Akt-mTOR-p70S6K pathway.

In the previous section, we showed that c-Myc protein expression was reduced in cells expressing kindlin-3 targeting siRNA even when fibronectin was not added to the cell culture. Hence, we compared the activities of Akt and p70S6K between different groups of cells in the absence of fibronectin and with or without serum starvation. No significant difference either in the level of phosphor-p70S6K or phosphor –Akt between wild-type cells, control siRNA cells and kindlin-3-siRNA(#149) cells was observed (Fig. 6f). Taken together, these data suggest that although kindlin-3 is involved in the regulation of c-Myc protein expression, the regulation appears to be independent of integrin α5ß1 and Akt-mTOR-p70S6K signaling pathways.

## Discussion

In this study, we show for the first time that kindlin-3, a well-established component of integrin signaling pathway, associates with RACK1-ribosome complex. Co-immunoprecipitation assays demonstrated that this association can be detected in immune cells and HUVECs. Data from *in vitro* ribosome binding experiments suggest that kindlin-3 associates with ribosome via RACK1 as the bridging molecule. The association of kindlin-3 with RACK1 and its complex formation with the ribosome were further verified by PLAs in HUVECs. Interestingly, we did not detect significant proximity signal between kindlin-2 and RACK1, suggesting that the association of kindlin-3 with RACK-1-ribosome is specific. This is in line with our co-immunoprecipitation studies using HUVECs and transfected 293T cells. The difference in interaction partners between kindlins is not unexpected given that they are functionally non-redundant. For example, although kindlin-3 and kindlin-2 are both found in the cytoplasm of HUVEC, only kindlin-2 is localized to mature focal adhesions^6^. siRNA-mediated silencing of kindlin-3 in HUVECs abrogated cell spreading and tube formation despite normal expression of kindlin-2^6^. Recently it was reported that kindlin-2 but not kindlin-3 interacts with high-affinity integrin-linked kinase (ILK)^46^. Hence, functional specialization of kindlins as a result of sequence variations along with different expression patterns, possibly provides cells tissue-specific adaptions to their micro-environments^47^.

**Figure 7:**
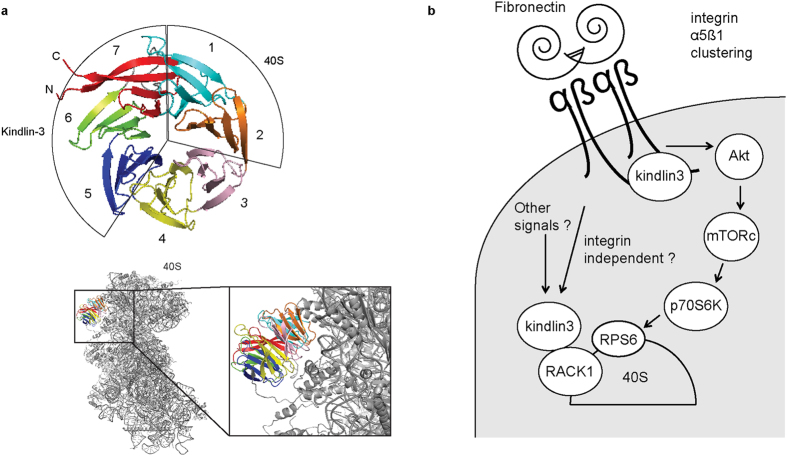
Illustrations. (**a**) The blades of RACK1 that are involved in binding ribosome 40S subunit and kindlin-3 are indicated. Illustrations were generated using the software Pymol (www.pymol.org) and the *S. cerevisiae* ribosome structural data (PDB ID: 3U5B and 3U5C)^40^. (**b**) Possible role(s) of kindlin-3 in protein translation and integrin α5ß1 signaling. In cells expressing kindlin-3, there are likely two populations of kindlin-3. One population associates with ribosomes via RACK1 and the other population regulates integrin avidity and its downstream Akt-mTOR-p70S6K signaling axis.

We have previously shown that blades 5–7 of RACK1 interact with kindlin-3^24^. Given that blades 1 and 2 of RACK1 interact with the 40S ribosome subunit^40^, the association of kindlin-3 with RACK1 on ribosome should not pose steric constraints based on available structural data on ribosome-bound RACK1 (Fig. 7a). RACK1 is located on the head of the 40S subunit close to mRNA tunnel exit site and has been shown to regulate protein synthesis^25^,^36^,^48^. Others have shown RACK1 promotes translation of survival proteins cyclin D1, c-Myc, survivin, and BCL-2 in chemoresistant hepatocellular carcinoma^34^. Here we found that silencing of kindlin-3 expression in human CML cell line K562 leads to a reduction in c-Myc protein expression. In line with this observation, both protein and mRNA expression levels of cyclin D1 were reduced in these cells. Consequently, we observed a decrease in proliferation rate of these cells *in vitro* and tumor formation capability *in vivo*. A role for kindlin-3 in cancer progression has been reported^21^,^22^. Sossey *et al.,* reported that kindlin-3 positively regulates breast cancer tumor growth^21^, whereas Djaafri *et al.,* observed a tumor suppressor role of kindlin-3^22^. What accounts for these opposite observations remains to be clarified. Our data suggest that kindlin-3 positively regulates CML K562 tumor growth. Although there was less vascularization in kindlin-3 siRNA tumor based on CD31 western blotting and kindlin-3 siRNA cells expressed and secreted lower level of VEGF-A, we rationalize that the smaller tumor growth of these cells is attributed primarily to the reduced c-Myc protein expression because smaller cell colonies were seen in the soft agar assays that is not dependent on either VEGF or vascularization.

Our data also showed that protein expression but not gene transcription of c-Myc was affected by kindlin-3 silencing in K562 cells. Kindlin-3 is involved in integrin outside-in signaling^24^,^49^. Integrin outside-in signaling is known to activate mTOR pathway that promotes cell survival^50^. Hence, we examined if kindlin-3 is involved in mTOR signaling triggered by integrin α5ß1, the predominant fibronectin-binding integrin in K562 cells^45^. We showed that kindlin-3 is involved in fibronectin-engaged integrin α5ß1 activation of Akt and p70S6K, which are upstream regulator and downstream effector of the mTOR complex, respectively. These data also suggest that kindlin-3 functions upstream of Akt and p70S6K, which is expected since kindlin-3 binds integrin ß cytoplasmic tail and it is involved in integrin activation and clustering. However, we also observed that in the absence of fibronectin, there was no significant difference in the levels of activated Akt and p70S6K between kindlin-3 siRNA cells, control siRNA and wild-type cells. Because c-Myc protein expression was reduced in kindlin-3 siRNA cells and these cells exhibited slower proliferation rate in suspension culture and in soft agar colony formation assay, these data collectively suggest that kindlin-3 regulates c-Myc protein expression by a mechanism that is independent of integrin α5ß1-Akt-mTOR-p70S6K signaling. In fact, kindlin-3-RACK1-ribosome complex was detected by co-immunoprecipitation of K562 cell lysate under standard culture conditions lacking fibronectin, suggesting that integrin signaling is not critical for the association of kindlin-3 with RACK1-ribosome. RACK1 has been shown to interact with c-Src^51^,^52^ and PKCßII^34^. It is possible that the association of kindlin-3 with RACK1 affects the interaction of RACK1 with these kinases, which in turn regulates c-Myc protein translation.

Apart from cancer progression, interplay between integrins, kindlin-3 and RACK1 may also be important in viral infections. The ß1 and ß3 integrins are usurped by different viruses for cell invasion and it involves integrin-mediated cytoskeletal re-modelling in many cases^53^. In the context of hematopoietic cells, activated integrin α5ß1 has been shown to be a co-receptor for human parvovirus B19 infection of erythroid progenitor cells^54^. Recently, RACK1 has been reported to promote internal ribosome entry site (IRES)-mediated translation of hepatitis C viral proteins^37^. Hence, it will be interesting to determine in future studies if kindlins are involved in integrin-mediated viral uptake and viral protein translation.

In summary, we have provided data demonstrating the association of kindlin-3 with RACK1-containing ribosome. Our data suggest that kindlin-3 is required for CML K562 proliferation and tumor formation by regulating c-Myc protein expression. Kindlin-3 regulates integrin α5ß1 activation of the Akt-mTOR-p70S6K pathway but this signaling axis is apparently non-essential for the association of kindlin-3 with RACK-1-ribosome and the role of kindlin-3 in c-Myc protein translation. Taken together, we propose that there are two populations of kindlin-3 that function at different levels of translational control (Fig. 7b). Translational control is a pivotal mechanism in cancer progression^55^, hence future work investigating the detailed mechanism by which kindlin-3 regulates c-Myc protein translation could provide useful insights for the development of alternative strategies in treating CML.

## Methods

### Chemicals and reagents

All general/common chemicals and reagents were purchased from Sigma-Aldrich (St. Louis, MO), Merck (Darmstadt, Germany), and Bio-Rad Laboratories (Hercules, CA) unless stated otherwise.

### Cell culture, transfection and siRNAs

K562 (human chronic myelogenous leukemia), HEK-293T (human embryonic kidney), and THP-1 (human monocytic) cell lines (American Type Culture Collection, Manassas, VA) were cultured in either complete RPMI1640 or DMEM medium, both containing 10% (v/v) heat-inactivated (HI) Fetal Bovine Serum (FBS), penicillin (100 IU/ml), and streptomycin (100 μg/ml) (Hyclone, Logan, UT). The complete RPMI1640 medium was also supplemented with 1 mM sodium pyruvate. Human umbilical vein endothelial cells (HUVEC) were maintained in EGM^TM^-2 supplemented complete medium according to instructions from the supplier (Lonza Group Ltd, Switzerland). HEK-293T cells (4.5 × 10^6^) in 10 cm cell culture dish were transfected with expression plasmid (8 μg) using the polyethylenimine (PEI) (Sigma-Aldrich)-based method. Cells were harvested for subsequent assays 24 h post-transfection. Generation of K562 cells stably expressing control-siRNA or kindlin-3 targeting siRNA, using lentiviral-based green fluorescent protein (GFP)-reporter system, have been reported previously^24^. The lentiviral siRNA system was generated by and purchased from Applied Biological Materials Inc, Canada). The oligonucleotide sequences of the siRNAs are: 5′-GGGTGAACTCACGTCAGAA-3′ (control siRNA, catalog number LV015-G); 5′-TGGAGCAGATCAATCGCAA-3′ (kindlin-3 siRNA #149); 5′-ACTACAGCTTCTTCGATTT-3′ (kindlin-3 siRNA #758). Both kindlin-3 siRNA sequences have been reported previously^56^. Rescue experiments were performed by micropipette electroporation^57^ of kindlin-3 siRNA #149 K562 cells with either pCDNA3.1(+) HA-tagged-full-length kindlin-3 or kindlin-3PHΔ or empty vector. For the kindlin-3 expressing plasmids, silent mutations (underlined nucleotides) in the kindlin-3 cDNA that is the target site of siRNA#149 (capital letters) were introduced as indicated: 5′-gTC GAA CAA ATT AAC CGG AAg-3′. This mutated sequence encodes the same amino acids as wild-type sequence: Val^50^-Glu-Gln-Ile-Asn-Arg-Lys^56^.

### Expression plasmids and purification of recombinant proteins

Construction of pCDNA3.1(+) plasmids containing HA-kindlin-2, HA-kindlin-3, HA-kindlin-3PHΔ, and HA-kindlin-3F3Δ has been previously reported^24^. Full-length human RACK1 cDNA was cloned into the pNIC28-Bsa4 vector and the recombinant protein with an N-terminal His_6_-tag was purified as previously described^26^. Generation of recombinant kindlin-3 was kindly assisted by the Protein Production Platform and Dr. Carrillo D Ruiz (Nanyang Technological University, Singapore). Briefly, full-length human kindlin-3 cDNA was cloned into pFB-LIC-Bse vector using ligand independent cloning (LIC) technology to generate a N-terminal His_6_-tagged fusion protein with TEV digestion site, transferred to bacmid using the Bac-to-Bac system (Invitrogen^TM^ Life Technologies, Carlsbad, CA) for large-scale expression in Sf9 insect cells^58^. The recombinant protein was purified by Ni-sepharose affinity and size exclusion chromatography. Gib2 cDNA (kindly provided by Dr. C. Xue from Rutgers University, Newark, NJ) was cloned into pOPTHis-Lip expression plasmid (a gift from Dr. R. Williams MRC-LMB, Cambridge, UK) to generate a TEV-cleavable N-terminal His_6_-tag fusion protein. The plasmid was transformed into *Escherichia coli (E. coli)* BL21(DE3) strain (New England Biolabs, Ipswich, MA) and induced to express the recombinant protein by 0.5 mM IPTG. Preparation of Gib2 has been described^42^. Briefly, harvested cells were re-suspended in buffer (300 mM NaCl, 5 mM β-mercaptoethanol, and 50 mM Tris, pH 8.0) and lysed in a high-pressure homogenizer (GEA Niro Soavi, Italy). Cell lysate was cleared by centrifugation (20,000 rpm for 20 min) and affinity purification was performed using HisTrap HP (5 ml) column (GE Healthcare, Buckinghamshire, UK). Imidazole was used for step-wise protein elution. Gib2-containing fractions were pooled together and dialyzed overnight at 4 °C against buffer containing 50 mM NaCl, 5 mM β-mercaptoethanol, and 50 mM Tris, pH 8.0. Sample was loaded onto HiTrap Q (5 ml) column (GE Healthcare) and increasing NaCl concentration was used for protein elution. Gib2 containing fractions were pooled together and concentrated to 10 ml before loading onto HiLoad 26/60 Superdex 75 pg column (GE Healthcare). Gib2 protein appeared in a single peak. Corresponding fractions were pooled together, concentrated, and stored at −80 °C.

### Immunoprecipitation assays and Western blotting

The rat anti-kindlin-3 mAb clone 9 has been reported^24^. Immunoprecipitation assays were performed as previously described with modifications^24^. Briefly, K562 cells (1.5 × 10^7^) were lysed in 800 μl of lysis buffer (150 mM NaCl, 1% v/v Igepal (Sigma-Aldrich), and 10 mM Tris, pH 7.4) containing protease inhibitor cocktail on ice for 30 min. For HUVEC immunoprecipitation experiments, 2.5 × 10^6^ cells were lysed in 500 μl of lysis buffer. The lysate was pre-cleared with 20 μg of rat serum IgG (Sigma-Aldrich) and 100 μl rabbit anti-rat IgG-conjugated protein A Sepharose (GE Healthcare) bead suspension. The lysate was then separated into two portions of same volume and immunoprecipitation was performed with mAb clone 9 (20 μg) and rat serum IgG (20 μg) with 100 μl rabbit anti-rat IgG-conjugated protein A Sepharose bead suspension at 4 °C for 3 h. Immunoprecipitated proteins were resolved on a 12% SDS-PAGE under reducing conditions and electro-transferred onto PVDF membrane (Bio-Rad). Western blots were performed using anti-kindlin-3 mAb clone 9 and primary antibodies from different sources following manufacturers’ instructions. Anti-RACK1 (mouse mAb B-3) and anti-RPL22 (rabbit mAb D-7) were from Santa Cruz Biotechnology (Santa Cruz, CA). Anti-RPS6 (rabbit mAb 5G10) was from Cell Signaling Technology (Danvers, MA). Proteins were detected using relevant horseradish peroxidase (HRP)-conjugated secondary antibodies (GE Healthcare) and ECL kit (Advansta Corp, Menlo Park, CA). Immunoprecipitation assay using anti-kindlin-3 rabbit polyclonal (pAb) antibody (Sigma) was performed as above with modifications. Pre-clearing step was performed using 15 μg of rabbit serum IgG (Sigma-Aldrich) and 100 μl of unconjugated protein A Sepharose bead suspension. 15 μg of either anti-kindlin-3 pAb or rabbit serum IgG was then used in the immunoprecipitation step. The same anti-kindlin-3 pAb was used to immunoblot kindlin-3. Immunoprecipitation assay was also performed using 8 μg of mouse anti-kindlin-3 (mAb 3D6) (Abcam) and 100 μl of unconjugated protein A Sepharose bead suspension. In this assay, 8 μg of mouse serum IgG (Sigma-Aldrich) was used as control. For immunoprecipitation experiments using 293T transfectants, 4.5 × 10^6^ cells were lysed in 500 μl of lysis buffer. Lysate was pre-cleared using 6 μg of mouse serum IgG and 100 μl of rabbit anti-mouse IgG protein A Sepharose bead suspension. This was followed by immunoprecipitation using 6 μg each of mouse serum IgG or mouse anti-HA mAb (Millipore Corp, Billerica, MA) and 100 μl of rabbit anti-mouse IgG conjugated protein A Sepharose bead suspension were used.

Western blotting of p70S6K and Akt and their activated forms were performed using the following primary antibodies from Cell Signaling Technology (Danvers, MA): rabbit anti-p70S6K, rabbit anti-phospho-p70S6K, rabbit anti-Akt, and rabbit anti-phospho-Akt (Ser473)(736E11). To examine the activation states of p70S6K and Akt under serum starved condition, cells were re-suspended in Hank’s balanced salt solution without glucose for 3 h under culture conditions. The cells were then lysed in lysis buffer containing protease inhibitors (Roche) and phosphatase inhibitor cocktail (Nacalai Tesque, Japan) for 30 min on ice. Total protein concentration was determined using bicinchoninic acid assay kit (Pierce, Thermo Scientific, Rockford, IL). Proteins were resolved by SDS-PAGE under reducing conditions. Western blotting of phosphorylated p70S6K and Akt were performed. The membranes were then stripped and re-blotted for total p70S6K and Akt. To examine the contribution of ligand-engaged integrin α5ß1 signal to Akt-mTOR-p70S6K pathway, cells were serum starved for 2.5 h, and then treated with or without 1 μM rapamycin (Sigma-Aldrich) for 1 h followed by incubation in serum-free medium containing human fibronectin (20 μg/ml) (Sigma-Aldrich) and MnCl_2_ (1 mM) for 15 or 30 min under culture conditions. For the 0 min time point, cells without fibronectin treatment were used. Densitometry measurements of Western blot protein bands were performed using the ImageJ software (open source, imagej.net). Other primary antibodies used in this study were: mouse anti-c-Myc (9E10) (Santa Cruz), mouse anti-cyclin D1 (Santa Cruz), rabbit anti-GAPDH (Cell Signaling Technology), and rabbit anti-CD31 (Abcam).

### Proximity ligation assay (PLA)

HUVEC (1.6 × 10^5^) were settled onto coverslip glass-bottom culture dish (MatTek Corp, Ashland, MA) and cultured overnight. Adherent cells were fixed in 3.7% (w/v) paraformaldehyde in phosphate buffered saline (PBS) for 10 min at room temperature (RT). Cells were permeabilized with 0.3% (v/v) Triton X-100 in modified cytoskeleton stabilization (CSK) buffer (100 mM NaCl, 300 mM sucrose, 3 mM MgCl_2_, 1 mM EGTA, and 10 mM PIPES, pH 6.8) for 1 min at RT and non-specific sites were blocked by incubating cells in PBS containing 1% (w/v) BSA for 30 min at RT. The permeabilized cells were incubated in PBS containing two primary antibodies (1 μg each) followed by Duolink *in situ* PLA (Sigma-Aldrich) anti-mouse (minus) and anti-rabbit (plus) probes, and detection reagent Red according to manufacturer’s instructions. The primary antibodies used for the first set of PLA experiments were IgG from rabbit serum (Sigma-Aldrich), rabbit anti-kindlin-2 (pAb)(Abcam), or rabbit anti-kindlin-3 (pAb) (Sigma) with mouse anti-RACK1 (mAb B-3) (Santa Cruz). For the second set of experiments, the primary antibodies used were IgG from mouse serum (Sigma), mouse anti-kindlin-3 (mAb 3D6) (Abcam), or mouse anti-RACK1 (mAb B-3) with rabbit anti-RPS16 (pAb T-19)(Santa Cruz). Actin filaments were stained with Alexa Fluor® 488-conjugated –phallodin (Invitrogen Corporation, Carlsbad, CA). Image acquisition was performed on a Zeiss LSM 710 confocal laser scanning microscope equipped with a 63x/1.4 oil DIC objective (Carl Zeiss, Oberkochen, Germany). Images were processed and presented using the software ZEN 2012 (Carl Zeiss). Proximity ligation signal in cells was analyzed using the Blobfinder software (The center for image analysis, Uppsala University).

### Ribosome isolation and binding assay

*S. cerevisiae* wild-type strain JD1370 (*MATa trp1 ura3 leu2 PEP4::HIS3 NUC1::LEU2*) (provided by Prof. Dinman, University of Maryland, College Park, MD) cells were grown and ribosomes isolated as previously described^40^. *C. neoformans* var. *grubii* (serotype A) H99 (*MATα*) wild-type and *gib2* knockout strain in H99 background, are a kind gift from Prof. Xue C. (Rutgers University, Newark, NJ). In brief, *C. neoformans* was grown in 5 liters of YPD (2% glucose, 1% yeast extract, and 2% Bacto Peptone) medium at 30 °C with continuous shaking (200 rpm) until OD_600_ 0.6–0.8. Cells were pelleted, re-suspended in 2 ml of buffer A (50 mM NH_4_Cl, 10 mM magnesium acetate, 100 mM EDTA, 5 mM DTT, 0.2 mM PMSF,10% glycerol, and 50 mM Tris, pH 7.0), and subjected to glass-bead homogenization (Precellys 24 Homogenizer, Bertin Technologies, France). All steps of ribosome purification and analyses were performed on ice or at 4 °C. Cell lysate was centrifuged at 50,000 rpm for 3 hours and the pellet was re-suspended in buffer B (buffer A with 500 mM KCl) followed by overlaying on a 25% glycerol cushion in buffer B and centrifugation at 50,000 rpm for 3 hours. The pellet was re-suspended in buffer A with 10% sucrose instead of glycerol and centrifuged at 5,000 rpm for 10 min to remove aggregates. The supernatant was diluted two-fold with buffer A (without glycerol), layered onto 10 to 30% sucrose gradient in buffer A and centrifuged at 19,000 rpm for 17.5 hours in SW 28 Ti type rotor (Beckman Coulter, Brea, CA). Ribosome profile was determined by continuous monitoring of A_260_ and 80S ribosome containing fractions were pooled together. Ribosomes were pelleted by centrifugation at 40,000 rpm for 20 hours and re-suspended in buffer G (50 mM KOAc, 10 mM NH_4_Cl, 5 mM Mg(OAc)_2_,2 mM DTT, and 10 mM HEPES, pH 7.5).

80S ribosomes (1.5 μM) were incubated with 6 μM kindlin-3 with and without 6 μM Gib2 or RACK1 in buffer G in final reaction volume of 25 μl at 30 °C for 25 min. For control, proteins were incubated in reaction buffer without ribosomes. Reaction mixture was filtered through Spin-X 0.22 μm filters (Corning Inc., NY). Reaction volume was increased to 50 μl with buffer G, layered onto 200 μl of 1.1 M sucrose cushion in the same buffer, and centrifuged at 45,000  rpm for 18 h. The pellet was washed once and dissolved in buffer G. Samples were resolved on a NuPage pre-cast 4–12% gel by electrophoresis.

### Ribosome sucrose density gradient fractionation assay

K562 and THP-1 cells expressing endogenous kindlin-3 were cultured in complete RPMI1640 media and treated with cycloheximide (100 μg/ml) for 30 min at 37 °C to arrest ribosome movement. Cells were pelleted, washed twice in fresh medium, and re-suspended in ice-cold lysis buffer (2.5 mM MgCl_2_, 1.5 mM KCl, 0.5% (v/v) Triton X-100, 0.5% (w/v) sodium deoxycholate, 1 mM DTT, and 5 mM Tris, pH 7.4) supplemented with RNase inhibitor (40U/μl). 100 μl of lysis buffer per 10 × 10^6^ cells was used. The cells were disrupted by up-and-down pipetting and the lysate was clarified by centrifugation at 13,000 rpm for 5 min at 4 °C. Subsequently the lysate (21 OD_260_ units) was loaded onto a 5 ml linear 5–45% sucrose gradient in buffer (100 mM KCl, 5 mM MgCl_2_, and 20 mM HEPES, pH 7.5) supplemented with 0.5 mg/ml heparin and 100 μg/ml cycloheximide. Ultracentrifugation was performed at 36,000 rpm for 1.5 h at 4 °C using a SW60-Ti rotor (Beckman Coulter, Brea, CA). Ribosome profile at A_254_ was monitored and collected as 250 μl fractions. Fractions were precipitated with ethanol and re-suspended in deionized water. RNA composition of fractions was determined by agarose gel electrophoresis and protein composition using 4–12% Nu-Page pre-cast acrylamide gel electrophoresis. Kindlin-3 and RACK1 were detected by Western blotting using appropriate antibodies as described above.

### Quantitative RT-PCR

Total RNA from cells was extracted using the Trizol (Invitrogen) method. Using 2 μg of total RNA as template, reverse transcription was performed for 1 h at 37 °C with the power SYBR Green Cells-to-CT kit (Ambion Life Technologies) according to the manufacturer’s protocol. Reaction was terminated by heating for 5 min at 95 °C. qPCR was performed using the KAPA SYBR fast universal qPCR kit (KAPA BIosystems) according to the manufacturer’s instructions on a CFX96™ Real-Time PCR Detection System (Bio-Rad Laboratories, Hercules, CA). The PCR conditions were: 95 °C for 10 min followed by 40 cycles of 95 °C (30 sec) and 58 °C (1 min). Melt-curves were assessed from 58 °C to 95 °C with an increment of 0.5 °C per 10 sec. The following primers synthesized by AITbiotech (Singapore) were used: kindlin-3 (forward) 5′-TTCCAGGCTGTGGCTGCCAT-3′ and (reverse) 5′-CCCAGCCAAGACAACCTTGC-3′; GAPDH (forward) 5′-GGTGAAGGTCGGAGTCAACG -3′and (reverse) 5′-CTCGCTCCTGGAAGATGGTG -3′; c-Myc (forward) 5′-CTTCTCTCCGTCCTCGGATTCT-3′ and (reverse) 5′-GAAGGTGATCCAGACTCTGACCTT-3′^59^; cyclin D1 (forward) 5′-CCGTCCATGCGGAAGATC-3′ and (reverse) 5′-ATGGCCAGCGGGAAGAC-3′; and VEGF-A (forward) 5′-GAAGTGGTGAAGTTCATGGATCTCTA-3′ and (reverse) 5′-TGGAAGATGTCCACCAGGGT-3′.

### Flow cytometry and cell proliferation assay

The surface expression level of integrin α5ß1 in control siRNA and kindlin-3 targeting siRNA producing K562 cells was determined by flow cytometry. Cells were incubated in PBS containing 20 μg/ml anti-integrin α5 (CD49e) antibody (Becton Dickinson, Franklin Lakes, NJ) for 1 h at RT. Thereafter, cells were washed in PBS and stained with allophycocyanin (APC)-conjugated goat anti-mouse secondary antibody (Becton Dickinson-Pharmingen) at a dilution of 1:400 at RT for 45 min. APC-conjugated antibody was used because the cells expressed the reporter GFP. Stained cells were analyzed on a FACSCalibur or Fortessa X20 (Becton Dickinson) and data are presented using the Flowjo software (Tree Star Inc., Ashland, OR). Cell proliferation assay in suspension culture was performed using the Cell Proliferation Dye eFluor® 670 (Affymetrix, eBioscience) according to manufacturer’s instructions. Briefly, cells (4 × 10^5^) were washed twice in PBS and re-suspended in 500 μl of pre-warmed PBS containing 5 μM of dye and incubated for 10 min at 37 °C in the dark. The labeling reaction was quenched by adding 5 volumes of ice cold complete RPMI1640 medium containing 10% (v/v) FBS. Cells were washed three times in the same medium and maintained under culture conditions. Cells were harvested every 24 h, fixed in 1% (v/v) formaldehyde in PBS, and stored at 4 °C before analyses by flow cytometry using the APC fluorescence channel.

### Soft agar colony formation assay

Soft agar (1% w/v Difco agar noble (Becton Dickinson) in deionized water) was mixed with an equal volume of 2X complete RPMI1640 medium at 40 °C. 320 μl of agar mix was added to each well of a 24-well culture plate and allowed to solidify for 30 min at RT. Cells were re-suspended in an equal volume of 0.6% soft agar mix at 40 °C to yield a final concentration of 4000 cells/ml. The cell-agar mix was layered on top of the solidified agar mix and allowed to solidify for 30 min at RT. Complete RPMI1640 medium was added on top of the agar to prevent desiccation and cells were grown for 14 days. Cell colonies were examined by microscope under a 4X objective with phase contrast (Olympus IX83 microscope, Japan). Cells were also stained with nitrotetrazolium blue chloride (NBT) (Sigma-Aldrich) overnight under culture conditions for clearer visualization of colonies. The area of tumor colony was determined using the CellSens Dimension software (Olympus).

### Xenograft tumor formation studies

K562 cells (expressing either control or kindlin-3 targeting siRNA) (5 × 10^5^) in 100 μl PBS were subcutaneously injected into the right flank of immune-compromised Balb/c-Rag-IL2Rγ null mice (kindly provided by K. E. Karjalainen, School of Biological Sciences, Nanyang Technological University, Singapore). After 28 days, the mice were sacrificed and the tumors were excised and weighed. Tumor volumes (V) were calculated by measuring the tumor length (l) and width (w) according to the formula: V = l × w^2^/2. Animal studies were conducted according to the Institutional Animal Care and Use Committee (IACUC) (Nanyang Technological University, Singapore) guidelines. All experimental protocols (ARF-SBS/NIE-A0252 and ARF-SBS/NIE-A0224) were approved by the Institutional Animal Care and Use Committee (IACUC), Nanyang Technological University, Singapore.

### ELISA for detection of secreted VEGF-A

Secreted VEGF-A was measured using the human VEGF-A Platinum ELISA kit (eBioscience). Briefly, cells (5 × 10^5^) were serum starved for 2.5 h, re-suspended in 1 ml of serum-free RPMI1640 medium, and settled into one well of a 12-well culture plate. Culture supernatant was collected after 8 h for ELISA detection according to manufacturer’s instructions.

### Shear flow analyses and electric cell-substrate impedance sensing (ECIS) measurements

Shear flow analyses were performed as previously described^49^, except that fibronectin-coated parallel-flow chips were used. Briefly, μ-slide I^0.4^ Luer flow chamber (Ibidi GmbH, Germany) was coated without or with fibronectin (5 μg/cm^2^) in PBS overnight at 4 °C thereby blocking non-specific binding sites in PBS containing 0.5% (w/v) BSA. The chip was placed on a microscope stage housed in a 37 °C custom-built plastic chamber. Cells were re-suspended in RPMI1640 culture medium (6 × 10^5^ cells/ml) with or without MnCl_2_ (1 mM) and infusion was performed using a syringe pump at a shear stress of 0.3 dyn/cm^2^. Bound cells in four different fields of the chamber were counted and the average number of cells per field was determined.

Real-time cell adhesion and spreading measurements were performed using the ECIS (Electric Cell-Substrate Impedance Sensing) system as described previously with modifications^24^. 50 μl of fibronectin in PBS (10 μg/ml) was dispensed into each well of a 16-well E-plate® device (Acea Biosciences, San Diego, CA) and incubated at RT overnight. The wells were washed in PBS and non-specific sites blocked in PBS containing 0.1% (w/v) BSA at RT for 30 min. K562 cells (8 × 10^4^) in 50 μl of culture medium were seeded into each well and AC impedance (cell index) was measured at 1 min intervals on a Real-Time Cell Electronic System ^TM^ (Acea Biosciences) placed in a humidified CO_2_ cell culture incubator. Function-blocking antibody (anti-CD49e, 10 μg/ml, Beckton Dickinson) and MnCl_2_ (1 mM) were included. Three wells (technical triplicate) were used for each condition and standard deviations (S.D.) were determined for every 10 min interval.

### Statistical analysis

Statistical analyses where appropriate are performed using the GraphPad Prism 5 software and described in the figure legends. p values are indicated in the figures.

## Additional Information

**How to cite this article**: Qu, J. *et al.* Kindlin-3 interacts with the ribosome and regulates c-Myc expression required for proliferation of chronic myeloid leukemia cells. *Sci. Rep.* **5**, 18491; doi: 10.1038/srep18491 (2015).

## Supplementary Material

Supplementary Information
