# Supplementary material for: Kindlin-3 interacts with the ribosome and regulates c-Myc expression required for proliferation of chronic myeloid leukemia cells
Source: Sci Rep. 2015 Dec 18;5:18491. doi: 10.1038/srep18491 (PMC4683439; doi:10.1038/srep18491)
Supplement: Supplementary Information [file srep18491-s1.pdf]

SUPPLEMENTARY INFORMATION

Kindlin-3 interacts with the ribosome and regulates c-Myc expression required for proliferation of chronic myeloid leukemia cells

Jing Qu[1+], Rya Ero[1+], Chen Feng[1], Li-Teng Ong[1], Hui-Foon Tan[1], Hui-Shan Lee[1], Muhammad HB Ismail[2], Wen-Ting Bu[1], Srikanth Nama[2], Prabha Sampath[2,3,4], Yong-Gui Gao[1,5*], and Suet-Mien Tan[1*]

[1] School of Biological Sciences, Nanyang Technological University, 60 Nanyang Drive, Singapore 637551, Singapore.

[2] Institute of Medical Biology, 8A Biomedical Grove, Singapore 138648, Singapore

[3] Department of Biochemistry, Yong Loo Lin School of Medicine, National University of Singapore 117597,Singapore

[4] Program in Cancer and Stem Cell Biology, Duke-NUS Graduate Medical School, Singapore 169857, Singapore

[5] Institute of Molecular and Cell Biology, 61 Biopolis Drive, Proteos, Singapore 138673, Singapore

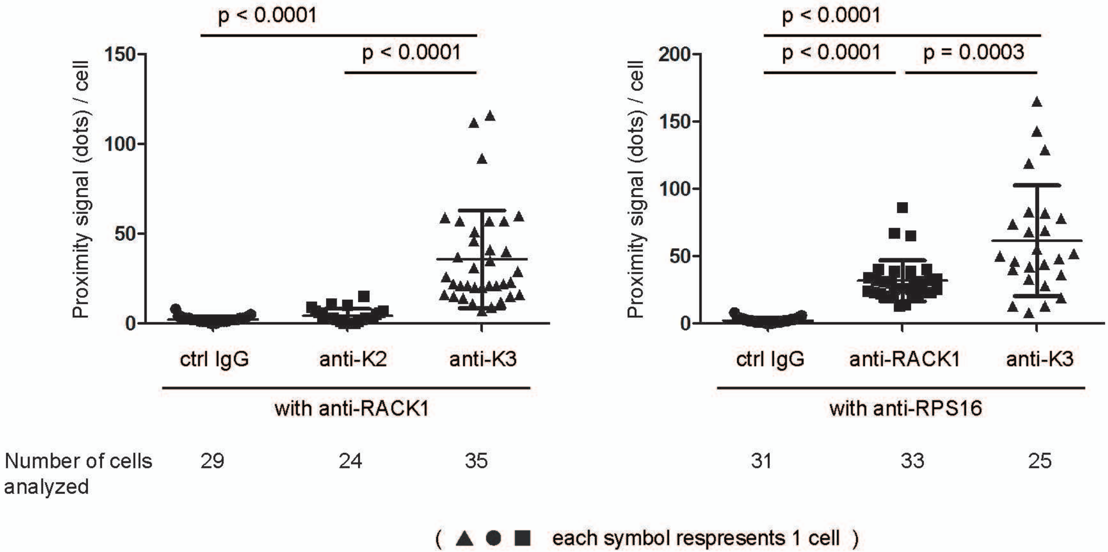

Supplementary Fig. S1. Proximity ligation assay (PLA) to detect kindlin-3 and RACK1-ribosome in HUVECs. Second independent experiment. Plots of number of red dots per cell are shown. Statistical analysis, two tailed unpaired *t* test.

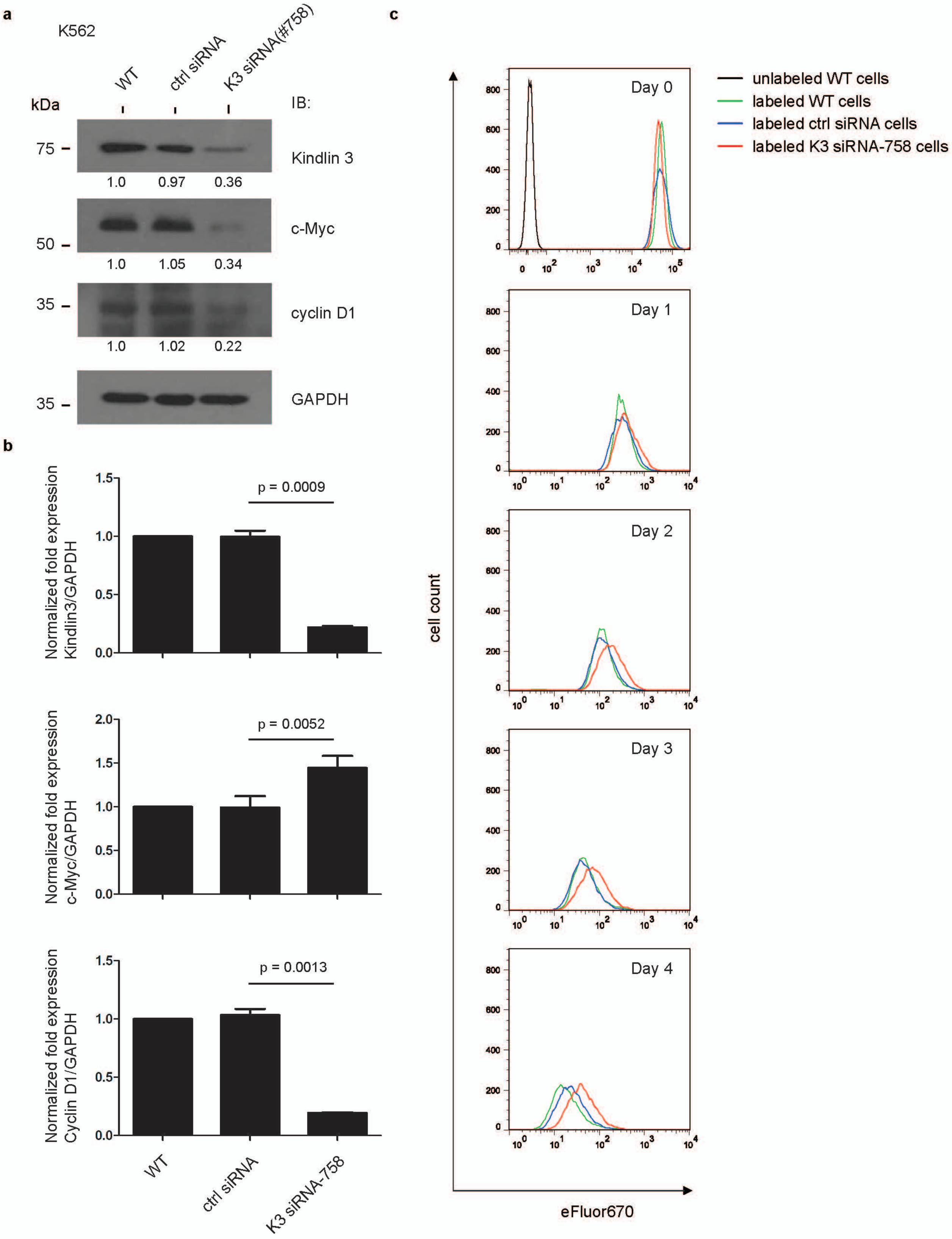

Supplementary Fig. S2. Kindlin-3 regulates c-Myc protein expression in K562 cells. (a) Western blot analyses of c-Myc expression in wild-type, control siRNA, and kindlin-3 siRNA#758 cells. GAPDH serves as loading control. Values below protein bands represent the mean fold differences in protein expression levels relative to WT samples (normalized to 1.0) from three independent experiments. (b) RT-qPCR analyses. Values represent mean ± S.E.M. of three independent experiments. Wild-type mean value was normalized to 1.0. Two-tailed unpaired *t* test was performed to compare control siRNA cells with kindlin-3 siRNA(#758) cells. $p < 0.05$ is considered significant. (c) Cell proliferation rate determination using cell proliferation dye eFluor® 670 and flow cytometry analyses. A representative experiment of three independent experiments is shown.

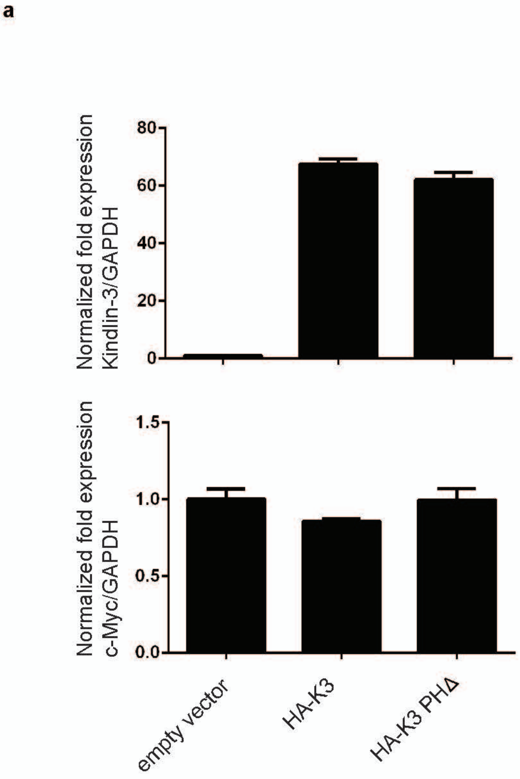

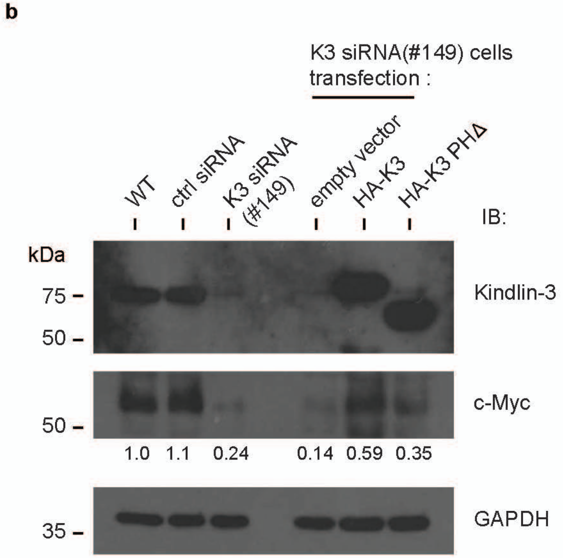

Supplementary Fig. S3. Rescue expression of kindlin-3 in kindlin-3 siRNA(#149) K562 cells restored c-Myc protein expression. (a) RT-qPCR analyses of kindlin-3 and c-Myc mRNA expression levels in kindlin-3 siRNA(#149) K562 cells transfected with either siRNA-resistant HA-kindlin-3 or HA-kindlin-3 PH deleted or empty vector. Data shown are mean +/- S.D. of technical triplicates.One representative experiment of two independent experiments is shown. (b) Western blot analyses of kindlin-3 and c-Myc. GAPDH serves as loading control. Wild-type cells, control siRNA and untransfected kindlin-3 siRNA(#149) cells were included as references. Values below protein bands represent the mean fold differences in protein expression levels relative to WT samples (normalized to 1.0) from two independent experiments.
